# Defining mental illnesses: can values and objectivity get along?

**DOI:** 10.1186/1471-244X-13-346

**Published:** 2013-12-24

**Authors:** Dominic Sisti, Michael Young, Arthur Caplan

**Affiliations:** 1 Department of Medical Ethics & Health Policy, Perelman School of Medicine, University of Pennsylvania, Pennsylvania, USA; 2 Harvard Medical School, Boston, USA; 3 Division of Medical Ethics, NYU Langone Medical Center, New York University, New York, USA

## Abstract

**Background:**

The creation of each edition of the Diagnostic and Statistical Manual (DSM) of psychiatry has proven enormously controversial. The current effort to revise the ‘bible’ of disorder definitions for the field of mental health is no exception. The controversy around DSM-5 reached a crescendo with the announcement from National Institute of Mental Health (NIMH) that the institute would focus efforts on the development of their own psychiatric nosology, the Research Domain Criteria (RDoC) (NIMH, 2013).

**Discussion:**

The RDoC seem to be structured around the concern that the only way to find objectivity in the classification of diseases or disorders in psychiatry is to begin with biology and work back to symptoms. Values infuse medical categories in various ways and drive practical considerations about where and how to divide up constellations of already agreed upon symptoms.

**Summary:**

We briefly argue that all nosologies are infused with values and, while we should continue to sharpen the psychiatric nosology, normativity will permeate even the strictest biologically based taxonomy; this need not be a bad thing.

## Background

The creation of each edition of the Diagnostic and Statistical Manual (DSM) of psychiatry has proven enormously controversial. The most recent effort to revise the ‘bible’ of disease definitions for the field of mental health was no exception. The controversy around DSM-5 reached a crescendo last April with the announcement from the National Institute of Mental Health (NIMH) that the institute would shift their efforts and funding to the development of their own psychiatric nosology, the Research Domain Criteria (RDoC), with hopes that this research will inform future iterations of the DSM and International Classification of Diseases (ICD) [1-4]. This announcement resulted in controversy and a flurry of media reports alleging NIMH was rejecting the DSM, prompting a joint statement of rapprochement by NIMH Director Tom Insel and Jeffrey Lieberman, who was president-elect of the APA at the time [5].

One source of the controversy is that the RDoC aims to classify mental disorders according specific functional analysis of particular genes, cells, neural circuits, and behaviors, arguably providing a higher degree of etiological validity and reliability than the current nosology. The goal will be to hone in on particular biological dysfunctions at various levels and link them to specific clinical phenomena. For example, the RDoC will jettison phenomenological approaches to the assessment of post-traumatic stress disorder favored by the DSM and ICD and instead focus on neural circuits implicated in fear-potentiated startle and other associated conditioned responses [6]. In so doing, varied disordered experiences of patients will be parsed into common underlying neural and genetic factors.

The Research Domain Criteria outlined by the NIMH appear to be structured around the concern that the only way to find objectivity in the classification of disorders in psychiatry is to begin with biology and work back to symptoms. The proposed methodology for this initiative thus specifies that “[t]he intent is to generate classifications stemming from basic behavioral neuroscience. Rather than starting with an illness definition and seeking its neurobiological underpinnings, RDoC begins with current understandings of behavior-brain relationships and links them to clinical phenomena” [1]. The proposal accordingly outlines a framework for research that is guided by “constructs” that “represent the fundamental unit of analysis in this system” and are “constrained by whether a particular brain circuit or area could reasonably be specified that implements that dimension of behavior”. These efforts align with the broader NIMH Strategic Plan to create “biosignatures” for mental disorders. The NIMH envisions that “a biosignature could consist of a genetic variant, an abnormal amount of a certain protein, a distinct neuroimaging pattern from a brain scan, a certain response during a cognitive test, or any number of indicators from blood, sweat, or other biological fluids” [2]. Concern is shared by other critics who worry that with disorders emerging involving sex, the internet, temper tantrums and shopping, the diagnosis of disorders of the mind will founder in the rough seas of subjective values and commercial interests.

## Discussion

Three key assumptions about the nature of mental disorders and about the role of biological data in explaining them appear to underpin the implementation of the RDoC and the aspirations that have motivated this pivotal transition. First, by setting out to devise a classification system based primarily on biomarkers and mechanisms, the RDoC assume that mental disorders are natural kinds that stand to be discovered and explained through a value-neutral blend of genetics, imaging, and neuroscience [3]. Second, in favoring this approach over that of the DSM, the RDoC suggest that a classification system based primarily on genetics, brain circuitry or other biomarkers could claim more validity than a classification system based primarily on traditional forms of clinical examination, clinical symptoms, patient history, phenomenology, or patient narrative. Finally, the RDoC envision that biomarkers can represent not only correlates of risk for mental disorder but may also constitute diagnostic tests for the presence or absence of a mental disorder, implying that any legitimate mental disorder has a potential biosignature or direct biological correlates. These assumptions spring from a deeper philosophical commitment that values and objectivity are incompatible.

Since significant consequences—financial, social, legal and medical—hinge upon decisions about classification, and since many critics inside and outside the field of mental health continue to insist that definitions of mental health, disorder, or disease are not trustworthy since they are fraught with subjective value judgments or lack biological foundations, it should not come as any surprise that controversy surrounds the appearance of DSM-5. What is rather surprising is the staying power of the view that ascribes “lack of validity” to any classification scheme that is not principally based in “objective laboratory measure” [4]. This view necessitates endorsement of the premise that reference to putatively “objective” (i.e., value-neutral) laboratory measures or biological data is the only path to validity within psychiatric nosology. This claim is intimately connected with a broader theory of disease-naturalism about mental disorders according to which psychopathology consists solely in disordered neurobiological mechanisms and value-free events that take place within the human organism. We would have thought that by now most researchers understand science and medicine to be a social enterprise, shot through with values be they molecular discoveries or clinical breakthroughs.

Values infuse medical categories in various ways [7]. Values can drive practical considerations about where and how to divide up constellations of already agreed upon symptoms. Or they might operate at a more fundamental level and influence what is considered to be dysfunctional or disordered behavior in the first place. For example, feminist philosophers have argued that political and gender-based values about “proper” feminine behavior have driven particular disorder categories such as histrionic and borderline personality disorders and premenstrual dysphoric disorder [8]. Lastly, values inevitably influence the clinical encounter, making diagnosis an exercise in medical hermeneutics by clinicians who must interpret self-reported symptoms of patients.

Many advocates of a strict biomedical model identify these features and wish to minimize the role of values in a classification of psychiatric disorders as they strive for what they consider to be a more legitimate classification scheme that “carves nature at the joints”. According to this naturalist view, scientific classification systems, be they about celestial objects, insect species, types of tumors, geological formations, or meteorological events, should reflect the observer-independent structure of the external world. To achieve this faithful capture of reality, those building classification schemes should strive to minimize any reference to or reliance on values [9]. Values, on this view, undermine objectivity.

For naturalists, the point of classification is to capture what is ‘out there’, value-neutral facts about the world that exist independently of those crafting the classification. Naturalism thus reflects the belief that there exists a Platonic world in which species, classes and kinds can be objectively distinguished by identifying their pure, essential properties without any reference to values [10,11]. On this view a valid DSM-5, liberated from values, and resting on a firm biological foundation would be accurate in all times and places. To put the point another way, a strict value-free, objectivist interpretation of a sound classification system of disease and disorder should be valid whether or not there are any humans interested in reading, hearing or using it.

This view of classification is, however, flawed. When critics argue that they find evidence of values shaping DSM-5 and in earlier editions of the DSM or ICD, they are right [7]. However, when they argue that such values undermine or invalidate the nosology, rendering objectivity impossible, they are wrong. The only way to understand the categories and disorder classifications to be found in psychiatric nosology—the DSM, ICD or one based on RDoC—is to acknowledge they are in part value-driven and value-laden [12,13]. A normative interpretation of nosology holds that values do not undermine objectivity; they are an essential component of objectivity in creating a guide to mental health and illness. Even the classification system advanced by the RDoC relies on normative, value-driven evaluation in deciding which behavioral characteristics constitute meaningful dysfunction and thus merit biological investigation, and in setting out to establish what degrees and dimensions of biological difference should be considered pathologic. Making inferences from biological differences to pathology and determining which problems should or should not be classified as ‘disordered’ is a project that cannot escape normative influence.

Classification systems, including nosologies, reflect the practical needs, values and interests of those doing the classification and, as in the case with DSM-5, input from key stakeholders [14,15]. What constitutes a hurricane as opposed to a tropical storm, a particular soil type or flood plain, a melanoma as opposed to a benign growth, are closely tied to human interests in traveling, farming and surviving. The fact that every classification reflects human interests and values or that DSM-5, in particular, reflects current human concerns and values does not undermine its objectivity or its validity.

We see the compatibility of values and objectivity in many cases in psychiatry. One example is borderline personality disorder, where, as we mentioned above, feminist concerns have convincingly shown that the diagnosis has been steeped in values. But this fact does not entail that the diagnosis is a whole-cloth social construction. On the contrary, the behaviors constituting the disorder are reliably observed across time and space, and these behaviors have a very real and generally negative impact on individuals and communities. That is to say, most would agree that the constellation of symptomatic behaviors of borderline causes suffering.

Therefore, unless consensus about values is impossible, the presence of values in motivating the creation of a classification or in shaping categories does not compromise the effort to secure an ‘objective’ nosology. Defining mental disorders requires both awareness and an incorporation of particularly important norms and values about human beings. Values such as self-determination, dignity, happiness and well-being serve as the pillars of psychiatric nosology. These core values, reflected in international treaties, legislation, conventions, religious and philosophical writings and judicial opinions, shape why and how various mental states, conditions, behaviors, drives and adaptations are classified as healthy or not. While every value is subject to debate and disagreement, both in terms of its meaning and the relative weight it should bear in a classification, consensus about the importance of core values such as self-determination and human dignity is not only possible, but exists. Consensus exists relative to what are key human rights, views about human flourishing, conceptions of well-being and the core functional requirements for daily living. DSM-5 reflects this current consensus.

DSM-5 reflects norms about human flourishing and human dignity that are linked to the importance of self-determination, the ability to exercise voluntary, free choice and to have opportunities to live life fully and in accordance with one’s personal values. Not only do these root the objectivity of DSM-5, it is almost impossible to imagine that a non-normative categorization of human mental health and illness could be achieved. Without values and norms, a book about human mental states and behavior would be a descriptive natural history and nothing more.

## Summary

The DSM-5 is a product of the desire to reduce human suffering and, as such, it reflects the prevailing, if evolving, values concerning what constitutes human flourishing. This does not reduce the disorders defined in the manual to mere tastes or preferences. Rather, it reflects the fact that core values permeate the creation of every nosology, that DSM-5 is no exception, and that a set of values suffuse particular decisions to continue, create or drop disorder categories. And this reality will be no different with RDoC.

The DSM-5 has been explicitly described and built to be a living document [16]. It will evolve as values shift and its precision will sharpen as knowledge of the mind and behavior and evidence about the efficacy of treatment continue to grow. It is precisely the contingency of all classification schemes in terms of values as well as the state of knowledge about the causes of behavior that leads to the need for ongoing revision, editing and updating of the DSM.

What is important is to not obscure or deny the role of values in formulating the DSM-5, but to be transparent about their role and to seek out input about the ways values and norms can be best captured by the manual. This is why consultation with many professional groups, patient advocacy organizations, and the general public was a key feature of the process of developing DSM-5. The values reflected in the nosology are not the product of who has the power to impose a value-laden perspective, but rather are the result of a commitment to reflect core social values about human dignity and capabilities that have been subjected to critique, conversation, and emendation by a wide and diverse audience.

Will RDoC eventually produce a more practical, accurate and less controversial psychiatric nosology? It could be decades before we know. But the idea that it will be somehow value-free and objective because it begins with genes instead of behavior is to impose a value on crude reductionism that will not lead to any more objectivity than can be found in the pages of the DSM-5.

## Competing interests

The authors declare that they have no competing interests.

## Authors’ contributions

All authors (DS, MY, AC) contributed to the planning, writing, and revision of the manuscript. All authors read and approved the final manuscript.

## Pre-publication history

The pre-publication history for this paper can be accessed here:

http://www.biomedcentral.com/1471-244X/13/346/prepub
